# Effects of acute physical exercise on oxidative stress and inflammatory status in young, sedentary obese subjects

**DOI:** 10.1371/journal.pone.0178900

**Published:** 2017-06-05

**Authors:** Francesca Accattato, Marta Greco, Salvatore A. Pullano, Ilaria Carè, Antonino S. Fiorillo, Arturo Pujia, Tiziana Montalcini, Daniela P. Foti, Antonio Brunetti, Elio Gulletta

**Affiliations:** 1 Department of Health Sciences, University “Magna Græcia”, Catanzaro, Italy; 2 Department of Medical and Surgical Sciences, University “Magna Græcia”, Catanzaro, Italy; Universita degli Studi di Catania, ITALY

## Abstract

Circulating oxidative stress and pro-inflammatory markers change after regular physical exercise; however, how a short session of acute physical activity affects the inflammatory status and redox balance in sedentary individuals is still unclear. Aim of this study is to assess antioxidant and inflammatory parameters, both at rest and after acute exercise, in sedentary young men with or without obesity. Thirty sedentary male volunteers, aged 20–45 (mean age 32 ± 7 years), were recruited, divided into 3 groups (normal weight: BMI < 25 kg/m^2^; overweight to moderate obesity: 25–35 kg/m^2^; severe obesity: 35–40 kg/m^2^), and their blood samples collected before and after a 20-min run at ~ 70% of their VO_2max_ for the measurement of Glutathione Reductase, Glutathione Peroxidase, Superoxide Dismutase, Total Antioxidant Status (TAS) and cytokines (IL-2, IL-4, IL-6, IL-8, IL-10, IL-1α, IL-1β, TNFα, MCP-1, VEGF, IFNγ, EGF). Inter-group comparisons demonstrated significantly higher Glutathione Reductase activity in severely obese subjects in the post-exercise period (P = 0.036), and higher EGF levels in normal weight individuals, either before (P = 0.003) and after exercise (P = 0.05). Intra-group comparisons showed that the acute exercise stress induced a significant increase in Glutathione Reductase activity in severely obese subjects only (P = 0.007), a significant decrease in MCP-1 in the normal weight group (P = 0.02), and a decrease in EGF levels in all groups (normal weight: P = 0.025, overweight/moderate obesity: P = 0.04, severe obesity: P = 0.018). Altogether, these findings suggest that in sedentary individuals with different ranges of BMI, Glutathione Reductase and distinct cytokines are differentially involved into the adaptive metabolic changes and redox responses induced by physical exercise. Therefore, these biomarkers may have the potential to identify individuals at higher risk for developing diseases pathophysiologically linked to oxidative stress.

## Introduction

Physical exercise induces metabolic changes in the organism, leading to the activation of adaptive mechanisms aimed at establishing a new dynamic equilibrium. One of the most significant changes in this regard occurs in the muscular tissue, in which the increasing energy demand following exercise generates a greater oxygen utilization by mitochondria [1]. Skeletal muscle is a major source of oxygen-free radical species because, during muscle contraction, an increased uncoupled transfer of electron from complex I and III in the electron transport chain leads to the production of superoxide radical (O^2-^), a primary member of reactive oxygen species (ROS). These toxic products of contractile activity are dismutated to hydrogen peroxide (H_2_O_2_) by superoxide dismutase (SOD), which is the first defence against radicals and, successively, detoxified by other enzymes such as catalase, glutathione peroxidase (GPX) and glutathione reductase (GR) [2,3]. A balance between ROS production and antioxidant enzyme expression and activity is crucial to sustain muscle redox homeostasis, and maintain ROS below a threshold level, and to keep their function as signaling molecules, while reducing their toxic effects [1]. On the other hand, ROS and anti-oxidant activity and capacity are correlated, so that these can be used as surrogate markers [4,5]. Blood antioxidant status may reflect the increase in oxygen demand in muscle tissue during physical exercise [6–8]. Therefore, circulating levels of these oxidative stress markers increase after both acute and regular physical exercise [8–10], probably as a mechanism of redox-mediated adaptation to protect against cellular oxidative damage [11]. However, the influence of different physical exercise protocols in the antioxidant balance has not yet been fully elucidated. Furthermore, while some investigations report that obese individuals have a greater increase in oxidative biomarkers after acute exercise in comparison to normal weight individuals [12,13], other studies show conflicting results [14,15]. Thus, whether and how people experience oxidative stress after an acute exercise is still debated.

Free radical generation leads to inflammation, lipid peroxidation and chromosome damage, which are associated with the onset of a wide range of pathological conditions, such as cardiovascular disease, diabetes mellitus, chronic obstructive pulmonary disease and cancer [16–19]. Oxidative stress is intimately related to and interconnected with hypoxia, a condition that is often exacerbated by increased oxygen radical production in a wide variety of diseases. Recent studies report an important role of hypoxia in the pathogenesis of obesity and obesity-related disorders, causing adipose tissue dysfunction, abnormal gene expression, and ultimately a systemic chronic, mild inflammatory state [20–22].

Physical exercise is known to be an important modulator of cytokines and chemokines, and for over a decade considerable attention has focused on the potential for exercise to prevent and treat diseases with an inflammatory component by modulating cytokine production. Although the positive effect of exercise on inflammation is well documented [23,24], the molecular mechanisms by which exercise exerts this effect are still unclear, and there is no consistent information in the literature about the variation of exercise-related markers. Several studies have reported that physical activity can induce an acute phase response characterized by an increase in multiple circulating cytokines and chemokines, such as Interleukin-1 (IL-1), Interleukin-6 (IL-6), Tumor Necrosis Factor α (TNFα), Epidermal Growth Factor (EGF), and Monocyte Chemoattractant-1 (MCP-1) [25–27]. However, other studies have shown no significant changes in cytokine levels after exercise [28–31], thus confirming that a lack of clarity exists on this issue, probably due to the complexity of the adaptive mechanisms during physical activity. Herein, we assessed the antioxidant and inflammatory status both at rest and after an acute, moderately intense exercise in sedentary, normal weight and overweight/obese young men, to test whether different early adaptive responses after a similar energy demanding stimulus may occur in these individuals. By using parameters that can be easily measured in clinical laboratories, we aimed to identify markers that could be potentially useful for patients at risk for diseases linked to oxidative stress.

## Materials and methods

### Enrollment criteria

Thirty male volunteers, aged 20–45, were recruited among the employees of the University Magna Græcia of Catanzaro. Women were excluded to avoid the evaluation of different hormonal states and specific hormonal response to physical exercise. All participants had a stable body weight during the preceding year. The inclusion criterion was sedentary lifestyle (i.e. not exercising for more than 20 minutes at least 3 days per week, based on self-report). None of the enrolled participants habitually participated in sport activity, were smokers or were taking medications or micronutrient supplements. We excluded individuals with clinical evidence of chronic, debilitating diseases (cancer, renal failure, liver dysfunction and pulmonary disease), thyroid dysfunction, individuals with weight loss surgery or undergoing weight loss program, and those with cardiovascular disease as based upon medical interview and physical examination. Written informed consent was obtained before enrollment. The following criteria were used to define cardio-metabolic risk factors: diabetes mellitus (fasting blood glucose ≥126 mg/dL or antidiabetic treatment); hyperlipidemia (total cholesterol > 200 mg/dL and/or triglycerides > 200 mg/dL or lipid lowering drugs use); hypertension (systolic blood pressure ≥ 140 mmHg and/or diastolic blood pressure ≥ 90 mmHg or antihypertensive treatment); body mass index (BMI) (overweight 25 ≤ BMI < 30 Kg/m^2^; moderate or class I obesity 30 ≤ BMI < 35 kg/m^2^; severe or class II obesity BMI ≥ 35 kg/m^2^); current smoking [32,33]. The protocol was designed according to the principles of the Declaration of Helsinki and approved by the Ethical Committee of the Azienda Ospedaliero-Universitaria “Mater Domini” of Catanzaro.

### Anthropometric and nutritional intake measurements

All tests were conducted after a 12-hour overnight fasting. Before tests, participants had no caffeinated beverages after meal and until the end of the test on the examination's morning. Body weight and height were assessed with a calibrated scale and a wall-mounted stadiometer, respectively, and BMI was calculated as the ratio: weight (kg)/height^2^ (m^2^). The nutritional intake of participants was calculated using the 24-hour recall method, in which information about all food and beverages used in the previous 24 hours were obtained. Information were analyzed with the nutritional software MetaDieta 3.0.1 (Meteda Srl, S. Benedetto del Tronto, Italy).

### Exercise protocol

Exercise was performed between 8:00 and 9:00 am, during which time enrolled individuals performed a run on an electronic treadmill (h/p/ cosmos T150, Nussdorf–Traunstein, Germany) for 20 min at approximately 70% of their VO_2max_, at a speed and grade set to keep the participants at their target heart rate (~80% of theoretical maximum heart rate (cHRmax), calculated as 220-age) [34]. No adjustments were needed for HRmax prediction on the basis of BMI [35]. Exercise intensity during the sessions was monitored by the use of a heart rate signal from a polar transmitter around the participant’s chest, until 3 minutes after recovery. All subjects were able to complete the exercise session.

### Measurement of biochemical parameters, oxidative stress markers and serum cytokines

Venous blood was obtained after fasting overnight and samples were immediately processed. Serum glucose, creatinine, total cholesterol, high density lipoprotein (HDL)-cholesterol and triglycerides were measured according to the manufacturer’s instructions, by Cobas 6000 (Roche, Switzerland) using the relative kits. Blood samples were also collected before and after exercise for the determination of the following indirect oxidative stress markers: GR activity and TAS in plasma, GPX activity in heparinized whole blood and SOD activity in erythrocyte lysate, according to the manufacturer’s instructions (reagents by Randox Laboratories Ltd, UK). Oxidative stress markers, whose assay principles are described below, were analyzed with the automatic RX Daytona Analyzer (Randox Laboratories Ltd, UK). SOD activity was determined by a method using xanthine and xanthine oxidase to generate superoxide radicals that react with 2-(4-iodophenyl-)-3-(4-nitrophenyl)-5-phenyltetrazolium chloride to form a red-formazan dye. GPX activity was assessed indirectly by a coupled reaction with GR, using a reagent set (RANSEL, RANDOX) [36], in which GR catalyzes the reaction whereby reduced NADPH converts oxidized glutathione (GSSG) to reduced glutathione (GSH). The oxidation of NADPH to NADP^+^ results in a decrease in the absorbance that is directly proportional to the GR activity. GR or GPX enzymatic unit (U) was defined as the amount of the enzyme that catalyzes the conversion of 1 micromole of substrate per minute. The definition of SOD enzymatic unit is the amount of protein that inhibits the rate of cytochrome C reduction by 50%. All enzyme activities were measured at 37°C. Circulating levels of cytokines and growth factors [Interleukin-1α (IL-1α), Interleukin-1β (IL-1β), Interleukin-2 (IL-2), Interleukin-4 (IL-4), IL-6, Interleukin-8 (IL-8), Interleukin-10 (IL-10), Interferon γ (IFNγ), TNFα, MCP-1, Vascular Endothelial Growth Factor (VEGF), EGF] were simultaneously measured using the “Cytokine Array I and High sensitivity”, based on a two-site chemiluminescent immunoassay, by the Randox Evidence Investigator analyzer (Randox Labs, UK), as previously described [37]. Quality control was daily assessed for all determinations, and results have met the precision targets indicated by the manufacturers.

### Statistical analysis

Data (see S1 Data) were expressed as mean values and standard deviation (Mean ± SD). The normal distribution of variables was analyzed by Kolmogorov-Smirnov test. In order to estimate the effects of confounding factors, a General Linear Model multivariate analysis was performed by incorporating age, and HDL-cholesterol as covariates (see S1 Table). Paired Student’s *t*-Test was used to compare means of continuous variables before and after physical exercise. ANOVA test was used to assess changes of antioxidant enzymes among the different BMI categories. Pearson's correlation test and regression analysis were performed to evaluate the correlation between clinical and laboratory parameters. A P value < 0.05 was considered statistically significant. Data were analyzed using the SPSS software version 20.0.

## Results

Demographic, anthropometric, and biochemical characteristics of the study population are shown in Table 1, as well as exercise parameters. Sedentary volunteers were divided into three groups according to BMI. Most of the clinical and biochemical characteristics were homogeneous in the three groups, although there were significant differences in the mean age, in calculated age-dependent parameters (cHR max and 80% cHR max), and serum HDL-cholesterol, with the last reflecting the known reduction occurring in obese individuals (Table 1). Although reporting bias could not be excluded, amounts of macronutrients and antioxidant micronutrients in the diet related to the 24-hour recall information were similar among groups, therefore these factors should not account for inter-group discrepancies in analytical results (Table 2).

**Table 1:** Baseline characteristics, biochemical parameters of the study population, and exercise-related indexes.

	Normal weight (n = 10)	Overweight/moderate obesity (n = 10)	Severe obesity (n = 10)	P value
Age (yrs)	26.50 ± 3	36.57 ± 7	32.14 ± 6	0.037
BMI (Kg/m^2^)	23.0 ± 1	28.4 ± 3	37.6 ± 3	< 0.001
Glucose (mg/dL)	83.8 ± 7	91.2 ± 11	98.5 ± 13	0.08
LDL-cholesterol (mg/dL)	103.8 ± 30	127.9 ± 32	123.7 ± 30	0.37
HDL-cholesterol (mg/dL)	59.8 ± 12	40.8 ± 10	45.1 ± 8	0.017
Triglyceride (mg/dL)	90.3 ± 67	188.3 ±125	105.0 ± 44	0.12
Creatinine (mg/dL)	0.98 ± 0.1	0.90 ± 0.1	0.85 ± 0.2	0.63
SBP (mmHg)	126.6 ± 5	126.6 ± 8	133.5 ± 9	0.21
DBP (mmHg)	81.6 ± 7	76.6 ± 12	85.7 ± 9	0.29
Basal HR (bpm)	74.0 ± 20	68.2 ± 8	72.8 ± 6	0.69
cHR max (bpm)	194 ±3	183 ± 7	188 ± 6	0.03
80% cHR max (bpm)	155 ± 2	147 ± 6	150 ± 5	0.03
3-min HR (bpm)	114 ± 2	113 ± 8	117 ± 2	0.94

Data are expressed as mean values ± standard deviation. The inter-group variability is determined by ANOVA test; significance level of P value is < 0.05. BMI, Body Mass Index; SBP, Systolic Blood Pressure; DBP, Diastolic Blood Pressure; HR, Heart Rate; cHR max, Calculated HR max; 3-min HR, 3-minute HR recovery; bpm, beats per minute.

**Table 2:** Amounts of macronutrients and antioxidant micronutrients in the diet related to the 24-hour recall information.

	Normal weight	Overweight/moderate obesity	Severe obesity	P value
Energy (Kcal/die)	1940 ± 409	1994.8 ± 515	2409 ± 800	0.28
Carbohydrates (g/die)	247.0 ± 52	235 ± 66	301.9 ± 90	0.47
Lipid (g/die)	72.3 ± 19	79.4 ± 28	100 ± 42	0.16
Protein (g/die)	73.8 ± 23	81.9 ± 26	89.9 ± 54	0.47
Selenium (μg/die)	33.7 ± 16	45.3 ± 23	37.4 ± 22	0.22
Vitamin C (mg/die)	119.8 ± 50	138.4 ± 64	162.1 ± 70	0.40
Vitamin E (mg/die)	13.9 ± 3	14.4 ± 4	15.1 ± 2	0.85

Data are expressed as mean values ± standard deviation. The inter-group variability is determined by ANOVA test; significance level of P value is < 0.05.

**Fig 1:**
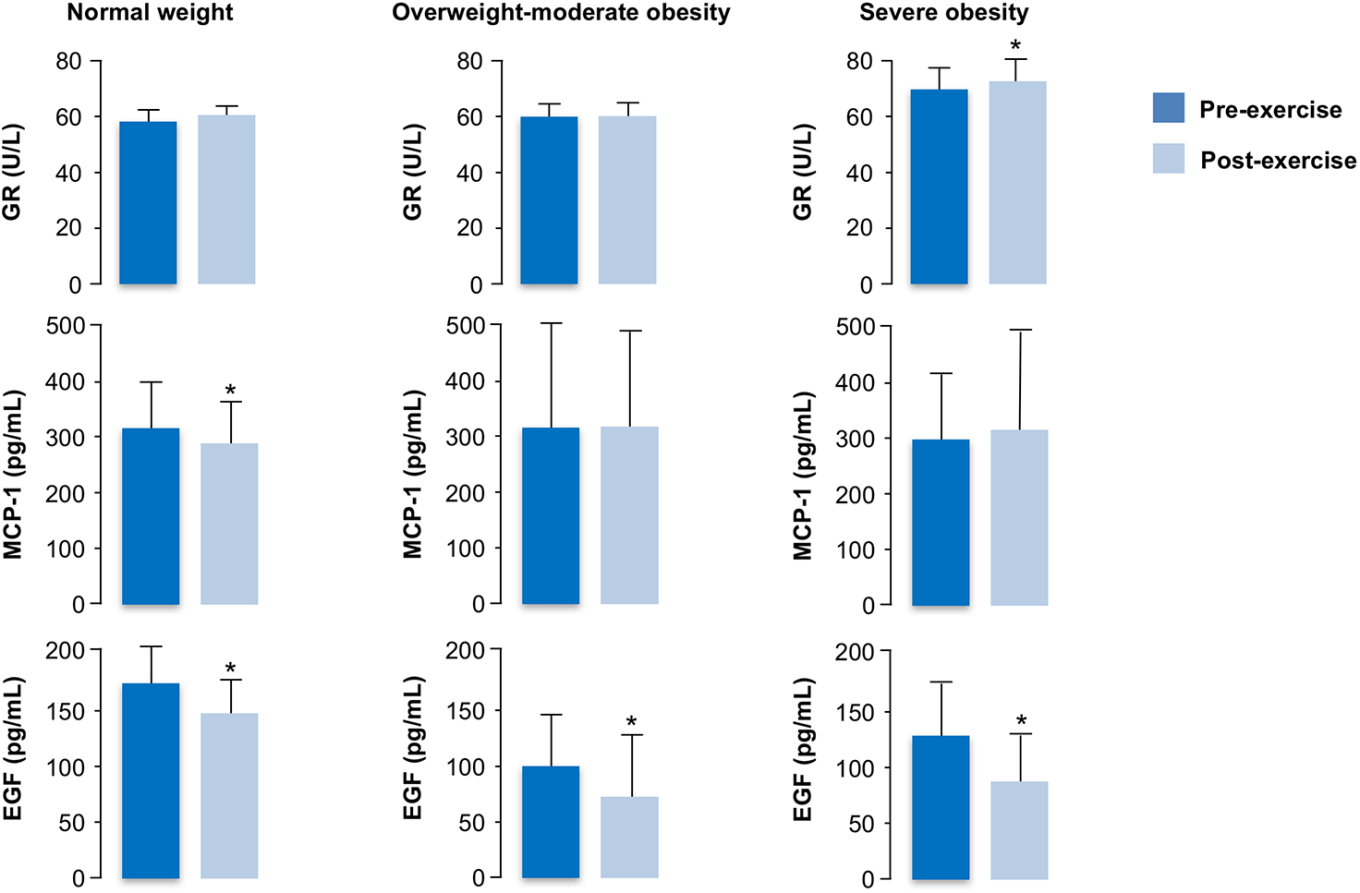
Pre- and post-exercise circulating GR, MCP-1, and EGF in the different BMI categories. Analytes were determined before and after 20 min exercise session at ~70% VO_2max_ in normal weight (BMI: <25 kg/m^2^, n = 10), overweight/moderate obese (BMI: 25–35 kg/m^2^, n = 10), severe obese (BMI: 35–40 kg/m^2^, n = 10) subjects. Data are expressed as mean ± SD.* denotes pre- vs. post-exercise statistical difference (P < 0.05).

When comparing the three groups of subjects, GR activity in the post-exercise period was significantly higher in subjects with severe obesity compared to those of other BMI categories (Table 3), even after adjustment for age and HDL-cholesterol (P = 0.036). When we considered data related to cytokines and growth factors, the results showed significantly higher levels of serum EGF both at rest (P = 0.003) and after exercise (P = 0.05) in normal weight individuals than in the other studied groups (Table 3). Statistical analysis of the results within the same group of obese persons, before and after exercise, indicated that among the oxidative stress parameters a significant increase of GR activity was detected after acute exercise in the severely obese group only (P = 0.007), with no statistical differences among the other groups (Table 4; Fig 1). In this context, a post-exercise decrease in MCP-1 level was observed in the normal weight group (P = 0.02), while no statistically significant variation of MCP-1 was observed after exercise in the other groups (Table 4; Fig 1). Interestingly, after performing the exercise session, EGF levels were significantly reduced in all BMI categories [normal weight (P = 0.025); overweight/moderately obese (P = 0.04); severely obese (P = 0.018)] (Table 4; Fig 1). In particular, a greater pre- to post-exercise percentage change was observed in severely obese (40%) compared to overweight/moderately obese (30%), and normal weight people (12%). No other significant differences were observed in serum cytokine levels among BMI categories and within the same group in relation to the physical stress (Tables 3 and 4), although TNFα and INFγ are close to significance in some intra-group comparisons (Table 4). Pearson’s correlation and regression analysis showed that BMI was positively correlated with post-exercise GR levels (*r* = 0.52, P = 0.017), and negatively with EGF (*r* = -0.49, P = 0.04).

**Table 3:** Circulating levels of oxidative markers and serum cytokines in the three BMI categories.

	Pre-exercise				Post-exercise			
Laboratory analytes	Normal weight	Overweight/moderate obesity	Severe obesity	P Value	Normal weight	Overweight/moderate obesity	Severe obesity	P Value
GPX (U/l)	318.5 ± 95	314.6 ± 120	316.3 ± 92	0.99	345.7 ± 97	261.1 ± 104	319.4 ± 86	0.29
GR (U/l)	56.4 ± 7	59.4 ± 7	67.6 ± 10	0.90	57.3 ± 4	59.3 ± 7	70.5 ± 12	*0.036
SOD (U/ml)	102.5 ± 30	88.8 ± 31	87.4 ± 49	0.74	114.3 ± 16	82.1 ± 47	104 ± 38	0.30
TAS (mmol/l)	1.93 ± 0.1	2.02 ± 0.2	1.93 ± 0.3	0.76	1.94 ± 0.1	1.98 ± 0.2	1.92 ± 0.2	0.89
IL-4 (pg/ml)	0.93 ± 1.4	1.41 ± 1.26	1.96 ± 1.2	0.78	2.61 ± 2.4	2.67 ± 1.65	0.89 ± 1.12	0.16
IL-6 (pg/ml)	1.29 ± 0.59	1.8 ± 0.4	1.83 ± 1.34	0.31	2.62 ± 2.24	1.93 ± 0.58	2.06 ± 1.81	0.25
IL-8 (pg/ml)	32.5 ± 20.3	19.3 ± 19.4	12.3 ± 12.9	0.26	34.4 ± 22.5	16.59 ± 10.9	17.32 ± 10.05	0.32
TNFα (pg/ml)	9.0 ± 2.7	10.17 ± 6.89	12.12 ± 9.34	0.76	7.37 ± 2.89	3.27 ± 3.97	3.63 ± 2.37	0.09
MCP1 (pg/ml)	313.8 ± 84.02	314.4 ± 182	299.4 ± 123.9	0. 97	281.2 ± 81.6	318.1 ± 164.1	326.1 ± 165.5	0.86
VEGF (pg/ml)	144.5 ± 145.9	144.9 ± 91.2	153.9 ± 106.8	0.99	114.9 ± 97.1	130.8 ± 75.8	170.1 ± 105.7	0.58
IFNγ (pg/ml)	8.43 ± 7.38	11.26 ± 16.21	8.87 ± 7.80	0.89	3.83 ± 3.5	3.56 ± 2.41	3.98 ± 3.51	0.97
EGF (pg/ml)	168.0 ± 37.4	99.72 ± 44.9	129.9 ± 37.5	0.03	147.8 ± 28.9	69.7 ± 60.8	80.8 ± 52.4	0.05

The inter-group comparison is shown before and after exercise. Data are expressed as mean values ± standard deviation. The inter-group variability is determined by ANOVA; significance level of P value is < 0.05.

*P adjusted for age and HDL-cholesterol.

**Table 4:** Circulating levels of oxidative markers and serum cytokines in the three BMI categories.

Normal weight				Overweight/moderate obesity			Severe obesity		
	pre-exercise	post-exercise	P value	pre-exercise	post-exercise	P value	pre-exercise	post-exercise	P value
GPX (U/l)	318.5 ± 95	345.7 ± 97	0.89	314.6 ± 120	261.1 ± 104	0.24	316.3 ± 92	319.4 ± 86	0.86
GR (U/l)	56.4 ± 7	57.3 ± 4	0.57	59.4 ± 7	59.3 ± 7	0.16	67.6 ± 10	70.5 ± 12	0.007
SOD (U/ml)	102.5 ± 30	114.3 ± 16	0.34	88.8 ± 31	82.1 ± 47	0.47	87.4 ± 49	104 ± 38	0.27
TAS (mmol/l)	1.93 ± 0.1	1.94 ± 0.1	0.23	2.02 ± 0.2	1.98 ± 0.2	0.40	1.93 ± 0.3	1.92 ± 0.2	0.43
IL-4 (pg/ml)	0.93 ± 1.4	2.61 ± 2.4	0.38	1.41 ± 1.26	2.67 ± 1.65	0.27	0.96 ± 1.2	0.89 ± 1.12	0.86
IL-6 (pg/ml)	1.29 ± 0.59	2.62 ± 2.24	0.93	1.8 ± 0.4	0.93 ± 0.58	0.48	1.83 ± 1.34	2.06 ± 1.81	0.30
IL-8 (pg/ml)	32.5 ± 20.3	34.4 ± 22.5	0.68	19.3 ± 19.4	16.59 ± 10.9	0.48	12.3 ± 12.9	17.32 ± 10.05	0.15
TNFα (pg/ml)	9.0 ± 2.7	7.37 ± 2.89	0.09	10.17 ± 6.89	3.27 ± 3.97	0.076	12.12 ± 9.34	3.63 ± 2.37	0.064
MCP1 (pg/ml)	313.8 ± 84.0	281.2 ± 81.6	0.02	314.4 ± 182	318.1 ± 164.1	0.79	299.4 ± 123.9	326.1 ± 165.5	0.31
VEGF (pg/ml)	144.5 ± 145.9	114.9 ± 97.1	0.22	144.9 ± 91.2	130.8 ± 75.8	0.26	153.9 ± 106.8	170.1 ± 105.7	0.31
IFNγ (pg/ml)	8.43 ± 7.38	3.83 ± 3.5	0.09	11.26 ± 16.21	3.56 ± 2.41	0.31	8.87 ± 7.80	3.98 ± 3.51	0.95
EGF (pg/ml)	168.0 ± 37.4	147.8 ± 28.9	0.025	99.72 ± 44.9	69.7 ± 60.8	0.04	129.9 ± 37.5	80.8 ± 52.4	0.018

The intra-group comparison is shown before and after exercise. Data are expressed as mean values ± standard deviation. The intra-group variability is determined by *t*-Test. Significance level of P value is < 0.05.

## Discussion

It is well known that exercise induces oxidative stress by an early increase of ROS, that acts, in turn, as a modulator of muscle and systemic adaptations to physical activity [38,39]. Although SOD is described as the first important shield against superoxide radicals, the glutathione system is, likewise, an important antioxidant defense. For example, there is evidence that the oxidative stress induced by hypoxia is primarily due to a decrease in GSH levels [40,41]. In this study, we found a significant and greater increase of GR activity after physical exercise in young, sedentary, severely obese volunteers, but not in overweight/moderately obese or normal weight subjects. Our findings therefore suggest that, in certain circumstances, even a short exercise session may be enough to activate antioxidant protection mechanisms against oxidative injury. In this context, we speculate that, when sedentary lifestyle and severe obesity are concomitant, an early antioxidant response may be required to resist the increased oxidative status that occurs following physical activity, whereas this does not occur in moderate obesity or sedentariness alone. Although sedentary lifestyle has been previously investigated by others [42], to our knowledge, this is the first study analyzing the oxidative stress marker GR in sedentary individuals with different ranges of BMI, in the context of a short run session. The choice of measuring oxidative stress markers after an acute bout of physical activity has been dictated by the need to assess early changes in pro-antioxidant balance under physical stress conditions, which might be not too different from the physical maximal stress to which obese subjects may be likely exposed during daily activities. Also, the same or a very similar exercise protocol has proved to be suitable to evoke changes in the oxidative balance and inflammatory status [43,44]. Although previous studies showed that physical exercise is an oxidant stimulus generating a ROS-dependent adaptive signaling [38,45], this effect seems to be affected by a high inter-individual variability [46]. In particular, recent studies showed that exercise-induced oxidative stress is exacerbated in obese subjects, in response to acute exercise [12–14,47]. Thus, our results are in line with previous findings in the literature. On the other hand, we were unable to test differences in oxidative stress markers across BMI status, although obesity, due to chronic overnutrition, sedentariness, as well as insulin resistance, has been associated with increased mitochondrial oxidative stress in insulin-target tissues, such as muscle and adipose tissues [48–50].

The mechanisms responsible for the variability in redox responses are still unclear and, at the same time, the answer to this question is crucial. Recent evidences support the concept that energy expenditure is controlled by a fine energy sensing network, that include PGC-1alpha, a key regulator of mitochondria, and two metabolic sensors, SIRT1 and AMPK that, in response to energy depletion or oversupply, may be tuned in opposite way, leading to metabolic fitness or complications [51]. In this regard, in our study, all volunteers were selected for being sedentary and assuming similar amounts of antioxidant micronutrients, two conditions that may affect the oxidative stress status [52]. High-calorie diet represents an underlying mechanism favoring free radical generation, and metabolic abnormalities in obesity, and, consequently, increased requirements of antioxidant enzymes under stress conditions [53]. However, other mechanisms can additionally explain the higher oxidative status in obesity.

It is well known that an increase in the endogenous antioxidant activity during acute exercise is a defense mechanism against hydrogen peroxide generation [54]. Several investigations link an increased oxidative stress to the abnormal activation of the renin-angiotensin-aldosterone system (RAAS) and multiple conditions are able to activate RAAS, including obesity. Furthermore, it has been shown that physical exercise can stimulate lipolysis [55], leading to increased levels of plasma glycerol and higher oxidative stress in obese than in lean subjects [56]. Thus, it is plausible that in severe obesity, the great lipid mobilization enhances the antioxidant requirement to limit free fatty acid oxidation.

In obesity, the hyperoxidative status is associated with a chronic low-grade inflammation, characterized by altered cytokine production with an increase of acute phase proteins and ROS [57–59]. Cytokines, in particular TNFα, IL-1 and IL-6, act as potent stimulators of ROS production by the monocyte-macrophage lineage cells infiltrating adipose tissue in obesity through the activity of NADPH oxidase [60].

Conflicting results have been reported about the effects of acute exercise on circulating cytokines levels. These discrepancies might be due to differences in the physical activity protocols applied (intensity, duration, and type of physical exercise) and to the choice of sampling time points which can mask the exercise-induced changes [61–63].

According to many scientific evidences, a single session of moderate aerobic activity, such as that undertaken by the enrolled individuals in our study, could not significantly change circulating cytokine levels [30–33]. Therefore, it is not surprising that, after the short physical exercise session performed in this study, serum levels of most of the cytokines analyzed remained unchanged. In our case, an early post-stress variation was only observed for MCP-1 and EGF, whose levels significantly decreased after the exercise, although, of note, also TNFα and INFγ show a reduction trend in some groups, coherently with previous findings that highlight the effects of physical exercise on the inflammatory response [43].

MCP-1 is a potent monocyte chemoattractant, mainly produced by monocytes, macrophages, and dendritic cells, which may induce adhesion molecule expression, tissue factor secretion, and smooth muscle cell proliferation in the context of inflammation, insulin resistance, and atherosclerosis. The adipose tissue is an important source for the release of MCP-1, which–by promoting the recruitment of immune cells to fat–contributes to inflammation and glucose intolerance in obesity [64]. Interestingly, at physiological concentration, MCP-1 is the only adipocytokine able to impair insulin signaling and glucose uptake in skeletal muscle [65]. Therefore, a decrease in this biomarker during physical exercise facilitates insulin-mediated glucose disposal. Although a decrease in MCP-1 even after a short exercise have been also reported by other studies [30,66], our results showed a post-exercise decrease in MCP-1 only in sedentary normal weight group, while no significant variation was observed either in overweight or obese people. We hypothesize that in obese subjects, due to a chronic low-grade pro-inflammatory status, a mild physical activity might not be sufficient to determine a reduction of this marker [67].

EGF is a potent activator of cell growth, proliferation and differentiation, and EGF receptors are highly expressed in adipose and muscle tissues [68]. EGF serum levels have been described to inversely correlate with fat mass and BMI, supporting a role of EGF in the pathogenesis of obesity and related diseases [69,70]. Consistently, we found a negative correlation between EGF and BMI, with significantly higher levels of EGF in normal weight than in obese subjects. After physical activity, we observed a significant reduction of EGF serum levels in all groups (although more evident in severely obese subjects). The same trend has been previously observed in young males after a similar exercise protocol [67]. A possible explanation for the decrease in EGF levels subsequent to physical activity may be linked to the role of EGF in ROS production. It has been reported that ROS may function as mediators of cellular responses, including EGF signaling pathway [71]. The EGF interaction with its receptor leads to the activation of NADPH oxidase and the consequent production of ROS, which, in turn, stimulate the autophosphorylation of the membrane receptor and the induction of the signal cascade [72]. It is plausible that the post-exercise EGF reduction, an already reported finding [67], is finalized to facilitate defense mechanisms against oxidative stress. This hypothesis is supported by the proportional reduction in EGF levels in association with increasing BMI, concurrently with the exacerbation of oxidative stress levels in obese subjects.

A particular strength of the present work is that the study has been performed in the same place, with the same conditions, while all the blood samples have been processed immediately to guarantee optimal analytical conditions.

Conversely, as a limitation of this study, the small sample size, due to the difficulty to enroll volunteers willing to have two consecutive blood samples withdrawn within a short time period. As a consequence of this limitation, the inter-group comparisons should be interpreted cautiously. In addition, as this investigation was performed in men only, caution should be used in generalizing conclusions.

## Conclusions

Based on our observations, this study may contribute to clarify some early physiological mechanisms by which sedentary individuals with different BMI categories respond to physical stress. We hypothesize that, while all BMI categories display a proportional reduction of EGF to limit ROS production, in severe obesity, GR activity increases to resist a more evident oxidative status. Concomitantly, in normal weight conditions, but not in obesity, a decrease in the pro-inflammatory cytokine MCP-1 facilitates insulin action in the muscle. All these observations are compatible with the concept that the adaptive mechanisms to physical stress may physiologically involve both the redox balance and the metabolic status. Understanding these mechanisms, and identifying novel biomarkers, may ultimately lead to new preventive and therapeutic strategies for obesity and obesity-related disorders.

## Supporting information

S1 Data Primary data. (XLSX)

S1 Table General Linear Model analysis. (DOCX)
