# Supplementary material for: Effects of acute physical exercise on oxidative stress and inflammatory status in young, sedentary obese subjects
Source: PLoS One. 2017 Jun 5;12(6):e0178900. doi: 10.1371/journal.pone.0178900 (PMC5459463; doi:10.1371/journal.pone.0178900)
Supplement: S1 Table — (DOCX) [file pone.0178900.s002.docx]

**General Linear Model Analysis.**

|  | **Raw Values** | | | | | |  | | **Adjusted Values** | | | | |
| --- | --- | --- | --- | --- | --- | --- | --- | --- | --- | --- | --- | --- | --- |
|  | | GR (U/I) post-exercise | | 95% CI | |  |  | | | GR (U/I) post-exercise | 95% CI | | Adjusted  P Value |
|  |  |  |  | Lower Bound | Upper Bound | P Value | |  | |  | Lower Bound | Upper Bound |  |
| Normal Weight | | 57.3 ± 4 | 54.19 | | 60.41 |  | |  | | 57.7 ± 3 | 50.35 | 65.12 |  |
| Overweight / moderate obesity | | 59.3 ± 7 | 53.95 | | 64.63 |  | |  | | 59.0 ± 3 | 52.19 | 65.81 |  |
| Severe Obesity | | 70.5 ± 12 | 62.04 | | 79.04 | **0.03** | |  | | 70.4 ± 3 | 64.54 | 76.26 | **0.036** |

Dependent Variable: GR (U/l) post-exercise. Age (yrs) and HDL-cholesterol (mg/dL) are included as covariates. Data are expressed as mean values ± standard deviation. (CI= Confidence Interval).
